# Post parotidectomy facial nerve palsy: A retrospective analysis

**DOI:** 10.12669/pjms.36.2.1706

**Published:** 2020

**Authors:** Atif Hafeez Siddiqui, Saad Shakil, Danish ur Rahim, Irfan Ahmed Shaikh

**Affiliations:** 1 Dr. Atif Hafeez Siddiqui, FCPS, Associate Professor of ENT. Department of ENT Head & Neck Surgery, Dow University of Health Sciences & Dr. Ruth K. M. Pfau, Civil Hospital Karachi, Karachi, Pakistan; 2 Dr. Saad Shakil, Resident PGY-4 ENT. Department of ENT Head & Neck Surgery, Dow University of Health Sciences & Dr. Ruth K. M. Pfau, Civil Hospital Karachi, Karachi, Pakistan; 3 Dr. Danish ur Rahim, FCPS, Assistant Professor ENT. Department of ENT Head & Neck Surgery, Dow University of Health Sciences & Dr. Ruth K. M. Pfau, Civil Hospital Karachi, Karachi, Pakistan; 4 Dr. Irfan Ahmed Shaikh, FCPS, Assistant Professor ENT. Department of ENT Head & Neck Surgery, Dow University of Health Sciences & Dr. Ruth K. M. Pfau, Civil Hospital Karachi, Karachi, Pakistan

**Keywords:** Parotid gland tumour, parotidectomy, facial nerve dysfunction

## Abstract

**Background &Objectives:**

Transient paralysis of facial nerve is seen to vary from 15 % to 66 % in post-parotid surgery. The objective of this study was to find out the complications in post-parotidectomy with regards to facial nerve dysfunction since it is a vital structure encountered in parotid surgeries.

**Methods:**

This was a retrospective study through non probability convenient sampling technique carried from September 2010 to January 2019 in the Department of Otorhinolaryngology, Dow University of Health Sciences, Dr. Ruth K.M.Pfau Civil Hospital, Karachi. Clinical data were recorded from 75 patients and out of them 70 patients had undergone surgery with parotid gland tumours and were reported on the morphology, age, sex, surgical procedure and complications, particularly facial nerve dysfunctions. In most cases ante-grade technique was performed to identify the facial nerve, whereas retrograde technique was used in recurring tumours, and in difficult cases. The stimulator of the nerve has not been used. The nature or severity of Facial nerve dysfunction was assessed in terms of either it is, permanent or temporary, total or incomplete in respect to its branches.

**Results:**

Among total 75 patients; the mean age was 38.75 ± 9.26 years with male to female ratio of 1:1. Majority of the patients were diagnosed as pleomorphic adenoma, i.e. 78.6% after which 12% were diagnosed as mucoepidermoid carcinoma. 88.6% of patients had superficial parotidectomy and 11.4% of patients had total parotidectomy. About 75% of patients had no complications. 5(7.1%) patients had complete facial nerve palsy. Damage to the mandibular, buccal and temporozygomatic branch was observed in 10(14%), 2(3%) and 1(1.4%) patients respectively.

**Conclusion:**

The most prevalent benign parotid tumour in this study was pleomorphic adenoma. After performing parotid surgery, it was predicted that the rate of complications related to the facial nerve injury was reduced as compared to the previous studies.

## INTRODUCTION

Tumours of the salivary gland account for approximately 3 % of total tumours while 5 % of the tumours of head and neck.^1^ Among the salivary gland tumours, 80 % belong to the parotid glands, 10 % to the submandibular glands while 10 % are present amongst sublingual gland as well as other minor salivary glands.^2^ Transient paralysis of facial nerve is seen to vary from 15 % to 66 % in post-primary parotid surgery, showing greater rate in total parotidectomy as compared to partial or superficial parotidectomy. Permanent paralysis of facial nerve is seldom seen and is only reported in 2.5 % to 5.0 % of cases. Parotid gland is a salivary gland consisting of deep and superficial lobes with facial nerve branches running between them.^3^ When the facial nerve emerges from the stylomastoid foramen, it passes over the postero-medial portion of the parotid gland for a shorter distance.^4^ Within the parotid, the division of facial nerve is seen as two main trunks i.e. the cervico-facial and the temporo-zygomatic branches, which also divides within parotid gland to form terminal branches. About 80% of the parotid gland tumours are of benign variety with majority of them i.e. 80 % being pleomorphic adenoma, followed by Warthin’s tumor and mono-morphic adenoma.^5^

Parotid gland’s superficial lobe is the area where majority of the tumours are found.^6^ Rapid rise in the size of the gland, pain and facial palsy are notable signs that signify cancer, sarcoidosis or tuberculosis.^7^ The only option of treatment in parotid gland tumour is surgical removal with two types of surgeries that are based on the location of tumour. For superficial lobe tumours, superficial parotidectomy is recommended and for tumours that arise from deep lobe, recurrent tumours and to preserve facial nerve in recurrent tumours, total parotidectomy is advised.^8^ Since facial nerve as well as its branches are very closely related to the parotid gland, trauma to the nerve is the chief concern during and after parotid gland surgery.^9^ Therefore, successful surgery of parotid gland is largely dependent upon the identification and preservation of the facial nerve. In tumours of the deep lobe and recurrent tumours, total parotidectomy is done which raises the risk of trauma to facial nerve during surgery. Facial nerve, especially due to marginal mandibular nerve damage is the most commonly encountered complication of parotid gland surgery.^10^ Temporary weaknesses of facial nerve after parotidectomy is also a common complaints wherein the expected time of recovery after surgery is approximately six months in most of the cases. After total parotidectomy. Facial nerve palsy is observed to be greater than in superficial parotidectomy, the reason being in relation to stretch injury or due to surgical trauma to vasa nervosum. Approximately in 8-46 % of benign parotid gland surgery, reports of neuropraxia are noted.^11^

The incidence of facial nerve dysfunction after surgery depends upon surgeon’s experience; however it is also dependent upon the size, differentiation as well as the location of the tumour. Inflammation or scarring of tissue, seen in recurrence or chronic disease has high susceptibility to trauma to the nerve during parotid gland dissection. Such aspect makes it hard for comparison or classification of surgical outcomes.^12^

The objectives of this study were to find out the frequency of facial nerve palsy and other complications related to facial nerve in patients undergoing superficial and total parotid gland surgeries.

## METHODS

This was a retrospective study through non probability convenient sampling technique carried from September 2010 to January 2019 in the Department of Otorhinolaryngology, Dow University of Health Sciences, Dr. Ruth K.M.Pfau Civil Hospital, Karachi.

Clinical data were recovered from 75 patients and out of them 70 patients were included in study who had undergone surgery with parotid gland tumours and were reported on the morphology, age, sex, surgical procedure and complications, particularly facial nerve dysfunctions. In most cases ante-grade technique was performed to identify the facial nerve, whereas retrograde technique was used in recurring tumours, and in difficult cases. The stimulator of the nerve has not been used. The nature or severity of Facial nerve dysfunction was assessed in terms of either it was, permanent or temporary, total or incomplete in terms of affected branches.

### Data Analysis

For data analysis, SPSS version 20.0 was used. Qualitative data was presented as frequency in percentage (%). Whereas quantitative data like age was presented as mean and standard deviation.

## RESULTS

Among the total of 75 patients selected for this study, male to female ratio was 1:1. The mean age was 38.75 ± 9.26 years. Majority of the patients were diagnosed as pleomorphic adenoma, i.e. 59(78.6%) and 9(12%) were diagnosed as mucoepidermoid carcinoma. Warthin’s tumor, cystadenoma, lymphoma and carcinoma ex pleomorphic adenoma were diagnosed in 01 (1.3%) patient each. [Table-I] 62(88.5%) of patients underwent superficial parotidectomy while total parotidectomy was done in 8(11.5%) patients. [Table-II] Out of the total 70 patients who underwent surgery, 52 (74.3%) patients were observed to have no complications while 5(7.1%) patients had complete but temporary facial nerve palsy. Damage to the mandibular, buccal and temporozygomatic branch was observed in 10(14%), 2(3%) and 1(1.4%) patients respectively. [Table-III].

**Table-I:** Classification of tumours in patients (n=75).

Type of tumour		n(%)
Benign	Pleomorphic Adenoma	59 (78.6%)
	Cystadenoma	1 (1.3%)
	Warthin’sTumour	1 (1.3%)
Malignant	Mucoepidermoid Carcinoma	9 (12.0%)
	Adenoidcystic Carcinoma	3 (4.0%)
	Carcinoma ex Pleomorphic Adenoma	1 (1.3%)
	Lymphoma	1(1.3%)

**Table-II:** Superficial and deep parotidectomy in patients (n=70).

Type of Tumour		n(%)
Total Parotidectomy	Mucoepidermoid Carcinoma	3 (4.28%)
	Deep lobe Pleomorphic Adenoma	5 (7.14%)
Superficial Parotidectomy	Pleomorphic Adenoma	54 (77.14%)
	Cystadenoma	1(1.42%)
	Warthin’s Tumor	1(1.42%)
	Mucoepidermoid Carcinoma (Low grade)	6(8.57%)

**Table-III:** Complications in Superficial and deep parotidectomy (n=70).

Complications		Total Parotidectomy	Superficial Parotidectomy	Total
Complete facial nerve Palsy	Permanent	0(0.0%)	0(0.0%)	0(0.0%)
	Temporary	3(4.28%)	2(2.85%)	5(7.14%)
Marginal Mandibular	Permanent	1(1.42%)	1(1.42%)	2(2.85%)
	Temporary	2(2.85%)	6(8.57%)	8(11.42%)
Buccal Branch	Permanent	1(1.42%)	0(0.0%)	1(1.42%)
	Temporary	0(0.0%)	1(1.42%)	1(1.42%)
Temporozygomatic	Permanent	0(0.0%)	0(0.0%)	0(0.0%)
	Temporary	0(0.0%)	1(1.42%)	1(1.42%)

## DISCUSSION

Our study showed that 88% of the patients underwent superficial parotidectomy while only 12% underwent total parotidectomy. Most cases, i.e. 78.6% of the patients were diagnosed as pleomorphic adenoma. Majority of the surgeries, 52(70%) had no nerve complications. However, Damage to the madibular, buccal and temporozygomatic branch was observed in 10(14%), 2(3%) and 1(1.4%) patients respectively

In a study by Shashinder S et al, facial nerve dysfunction was reported in 28% cases immediately after surgery.^13^ In a series of parotidectomies, Rehman et al observed 26.6% temporary facial weakness compared to Ramadan et al, who observed 34% transient facial weakness.^14^,^15^ Transient facial nerve dysfunction was noted by Adeyoma et al. in 30% of cases.^16^ On the contrary, El-Shakhs et al. observed temporary facial paralysis in only 16.6% of parotidectomies and after surgery.^4^ Surprisingly, the frequency of temporary facial weakness in our study has been reported at 8.5 % which is quite low with respect to most of the studies reported above. One of the study reported the rate of temporary facial weakness was 5 – 20%.^17^ Frequency of temporary and permanent facial nerve dysfunction was 23% lower in superficial parotidectomy than 49% in total parotidectomy and 65% in recurrent tumors. Multiple studies have reported the increased frequencies of facial nerve injury.^14^,^15^ In another study, 90% of cases of facial nerve injury involved the marginal mandibular nerve and 54% cases showed zygomatic.^14^ The finding of the above study is contrary to our study in which we observed only 14.2 % of cases involving the mandibular branch of facial nerve.

After anatomical preservation in parotid surgery, many theories have explained the facial nerve dysfunction. This can be caused by mechanical trauma, such as crushing and compression during surgery or ischemic injury during nerve dissection. Dulguerov et al. found that after anatomical preservation, nerve stretching could be the most possible aetiology of facial nerve dysfunction.^18^ The frequency of the facial nerve dysfunction may also be associated to nerve identification techniques, but recent evidence suggests no difference between ante grade and retrograde techniques in the rate of temporary nerve dysfunction.^19^

In yet another study by Huang G et al, in which among 79 patients that underwent partial superficial parotidectomy and 241 patients that underwent superficial parotidectomy, 6 (7.6%) and 55 (22.8%) patients in the respective group suffered immediate facial nerve paralysis having a significant difference of 0.003. Five patients from the partial parotidectomy group experienced Frey’s syndrome and 38 patients from superficial parotidectomy group experienced Frey’s syndrome having a significant difference of 0.03 between the groups.^20^ The findings of the above study are contradictory to our study in which the frequencies of facial nerve injuries were much reduced.

Musani MA et al, reported that among the total of 235 patients undergoing parotidectomy with 188 (80%) underwent superficial parotidectomy while 47 (20%) underwent total parotidectomy. Forty three (23%) of patients experienced facial nerve palsy in the superficial parotidectomy group while 23 (49%) patients in the total parotidectomy group experienced facial nerve palsy.^21^

Tung BK et al showed that in a cohort of six year follow-up, all their 54 patients of the study suffered immediate postparotidectomy facial nerve dysfunction with weakness of marginal mandibular branch and 07 % of patients also had co-existing zygomatic branch dysfunction. Majority, 45 (83%) of patents regained total nerve function and recovered well.^22^ These findings are in contrast to our study in which we observed a remarkable decrease in the incidence of facial nerve injuries.Surgeon’s experience and surgical techniques could possibly play a vital part in having a decreased occurrence of facial nerve dysfunction in post-parotidectomy patients.

### Limitations of the study

Retrospective nature of the study and observer bias are the elements of this study which are the limiting factors. Large scale, multi-centered, prospective studies involving different surgeons in different hospital settings would help to discover more facts on the complications after parotidectomy.

## CONCLUSION

The most prevalent benign parotid tumour in this study was pleomorphic adenoma. After performing parotid surgery, it was predicted that the incidence of complications related to the facial nerve injury were lesser as compared to the available literature. Furthermore, it is recommended that proper knowledge of anatomical landmarks and careful dissections may be helpful for reducing the rate of facial nerve injury after parotidectomy.

### Author’s Contribution:

**AHS:** Conceived, designed, supervised, and did review, approval of final manuscript, takes responsibility for integrity of research.

**SS:** Did data collection, statistical analysis and manuscript writing.

**DUR:** Did data collection and approval of final manuscript.

**IAS:** Did data collection and approval of final manuscript.
